# Knowledge, attitude and practice (KAP) survey of canine rabies in Khyber Pakhtunkhwa and Punjab Province of Pakistan

**DOI:** 10.1186/s12889-020-09388-9

**Published:** 2020-08-26

**Authors:** Touseef Ahmed, Sabir Hussain, Ubaid-ur-Rehman Zia, Sangay Rinchen, Ammar Yasir, Shafique Ahmed, Waqar Ali Khan, Muhammad Farooq Tahir, Robert Ricketson

**Affiliations:** 1 Department of Epidemiology and Public Health, University of Veterinary and Animal Sciences, Lahore, Pakistan; 2 Department of Biological Sciences, Texas Tech University, Lubbock, TX USA; 3 Regional Livestock Development Centre, Department of Livestock, Tsimasham, Chukha, Bhutan; 4 Centre of Excellence in Molecular Biology, Lahore, Pakistan; 5 Department of Clinical Medicine and Surgery, University of Veterinary and Animal Sciences, Lahore, Pakistan; 6 Health Security Partner, Washington, DC USA; 7 Hale O’mana’o Research , Edmond, OK USA

**Keywords:** Rabies, Infectious disease epidemiology, Zoonosis, KAP survey, Pakistan

## Abstract

**Background:**

This study aimed to assess the extent of knowledge and understanding of rabies disease in rural and urban communities of Pakistan. It also identified malpractices after suspected dog bite that might pose a risk for humans contracting rabies.

**Methods:**

A cross-sectional study was conducted (*n* = 1466) on people having different age groups and educational levels in four different geographic regions of Punjab and Khyber Pakhtunkhwa provinces in Pakistan. Knowledge, attitude, and practices of people were assessed using a structured questionnaire. We used a bivariate and multivariate analysis to study the association between rabies related mortalities in near or extended family members and different risk behaviors.

**Results:**

Our results demonstrate that the majority of the juvenile population (less than 18 years of age) were not aware of the clinical signs of rabies in animals. 75% of the total respondents were not vaccinated against rabies, 60% did not seek a doctor’s advice after a suspected animal bite, and 55% had inadequate health care facilities for rabies patients in local hospitals.

Respondents that had pets at home had not vaccinated (38%; *p* < 0.05; odds ratio 1.58) themselves against rabies due to lack of knowledge and awareness of pre-exposure prophylaxis for rabies (51%; *p* < 0.05; odds ratio 1.25). They also tend to not visit doctor after suspected bite (52%; *p <* 0.05; odds ratio 1.97), which may had resulted in more deaths (65%; *p <* 0.05; odds ratio 1.73) of someone in their near or extended family due to rabies.

**Conclusions:**

Lack of knowledge about the nature of rabies disease and prophylaxis has contributed to increase of rabies related deaths. Inadequate health care facilities and poor attitude of not seeking medical attention after suspected dog bite are the major reasons of rabies related deaths. These findings could help in devising a targeted management strategy and awareness program to control and reduce the incidence of human rabies related deaths in Pakistan.

## Background

Rabies is considered as one of the oldest infectious disease that affects all mammals [1, 2]. This disease is caused by a *rhabdovirus* and is usually transmitted to humans through the bite from a rabid animal [3]. The high burden of rabies associated mortalities in most developing countries like Pakistan, predict the existence of ineffective human and animal rabies prevention and control programs [4].

Rabies is endemic in Pakistan with over 50,000 reported cases of dog bites and approximately 6000 deaths annually resulting in huge economic losses [2, 4]. Globally, Rabies claims 55,000 deaths annually making it the 11th most deadly infectious disease worldwide. The highest burden of rabies associated mortalities are reported from subcontinents with over 30,000 deaths, followed by Africa [4]. The incidence density of human deaths linked with rabies typically ranges from 20 to 30 cases per million people yearly in India [5], 14 cases per million people yearly in Bangladesh [6] and 7.0 to 9.8 cases per million people yearly in Pakistan [7].

Knowledge, attitudes, and practice (KAP) surveys are widely used around the world for public health related studies based on the principle that knowledge will increase health seeking behavior and practices against disease. As a result of changing attitudes and practices disease burden can be minimized as seen in case of different disease conditions [8]. For instance in Thailand, a KAP survey influenced in increased community awareness on the control and prevention of dengue [9]. Similarly, KAP surveys identified knowledge gaps, behaviors, and cultural beliefs which may pose barriers to control infectious especially zoonotic diseases [10, 11]. KAP studies can be used for arranging public health awareness campaign and also result in provision of baseline data for planning, application, and evaluation of national diseases control programs [12]. KAP surveys have applied to study rabies in order to generate baseline data [11]. This baseline data in essential in tracing major loops in knowledge, awareness, and practices related with rabies for its control and prevention [11].

## Methods

### Study area

**Fig. 1:**
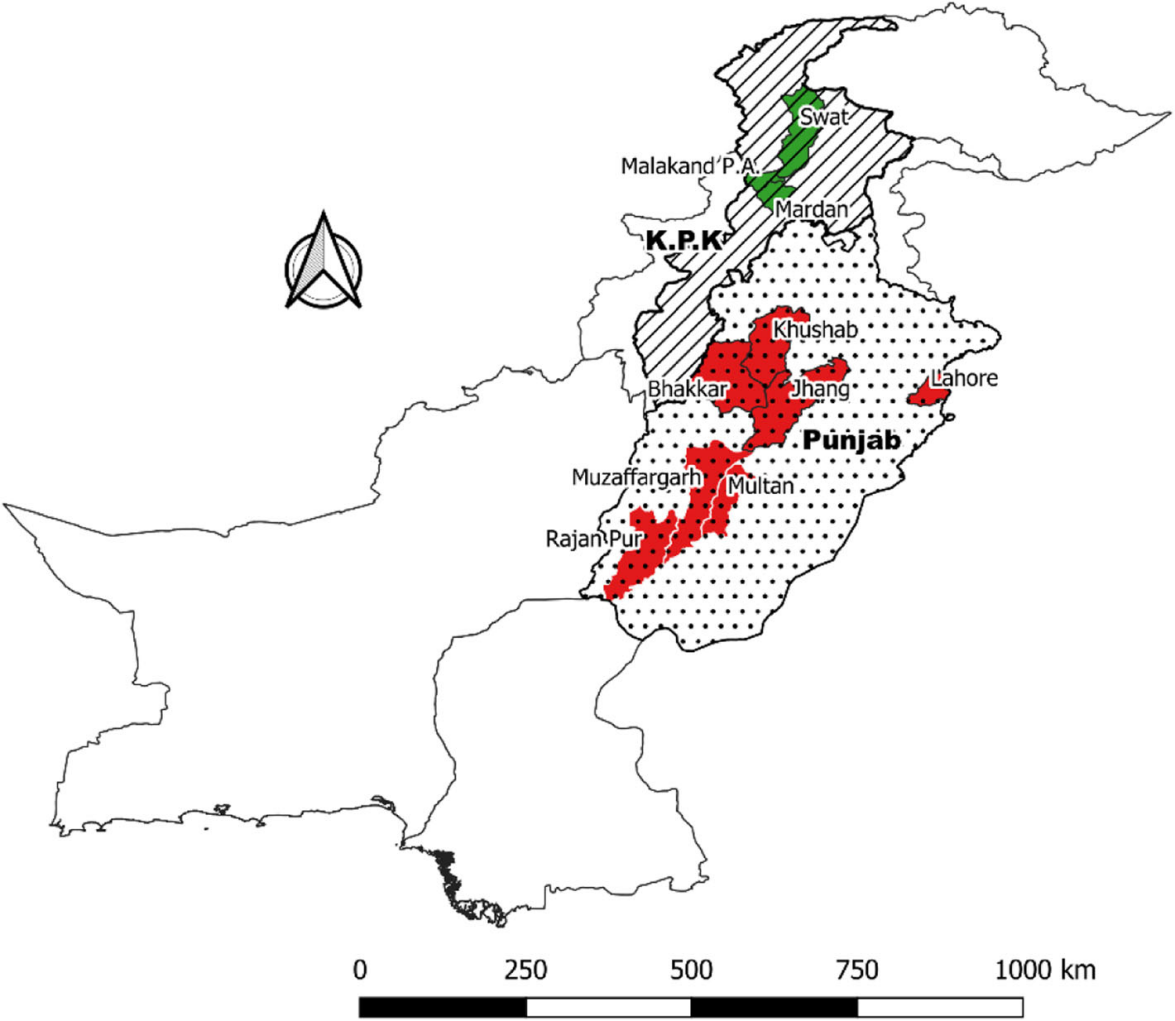
Map Representing Study Area. The map of Pakistan showing study districts where Knowledge, Attitude, and Practices KAP Survey on Rabies Disease was conducted. Study districts in Khyber Pakhtunkhwa and Punjab are shown by Green and Red Color respectively. The map was generated using open source Quantum GIS software specifically for the purpose of this study [13]. The shapefiles for the political boundary of Pakistan including district and sub-district boundaries were obtained from the Pakistan Bureau of Statistic without any specific approval [13]

Our KAP surveys were conducted in Punjab and Khyber Pakhtunkhwa formally known as KPK, province of Pakistan (Map.1.0). The study covered approximately 78,712 Km^2^, representing 8.92% of the country’s land mass. These areas are inhabited by about 33 million people that represent 15.5% of the entire population of Pakistan (Pakistan Bureau of Statistics, 2017). 10 districts were selected from these 4 regions (seven districts in Punjab; three districts in KPK). These study regions were selected because of their differences in topography and daily interaction with animals, both of which can contribute to rabies spread [Fig. 1]. Hilly region mostly hilly with an average elevation of above 5000 ft above sea level and mainly includes sampling areas from KPK Province while semi hilly region with an average elevation of 2000 to 5000 ft above sea level and mainly includes sampling areas around the border of Punjab and KPK province. The plains region includes areas of Punjab province with an average elevation of less than 700 ft above sea level. The desert regions in these provinces have an annual precipitation of below 220 mm. All surveys were conducted from September 2018 to January 2019 by 10 trained enumerators.

### Sampling techniques and sample size

Data was collected on a pre-designed, structured questionnaire from different sites in the 4 aforementioned geographic regions of Punjab and KPK province by trained enumerators. Convenient sampling technique was used to collect data regarding knowledge, attitude, and practices of rabies. For the sake of convenience, to maximize accuracy along with response rate, and to avoid any sort of confusion by the respondents, the questionnaire was translated into the local language in that region. Data was collected after obtaining informed verbal consent using debriefing form. Each respondent including guardian or parent of respondent below 16 year of age was informed using same debriefing form. Verbal consent is preferred because it is socially and culturally acceptable in comparison to written consent which creates lot of suspicion.

We initially assumed 50% of our respondents might have knowledge and awareness about transmission, clinical signs, and acceptable preventative practices against rabies. This survey included people of both genders, rural and urban locations, and different levels of education. Juveniles (less than 18 year of age) were surveyed as a focus group because they are reported to have the highest exposure rate to rabies disease [14] (Table 1).

**Table 1:**
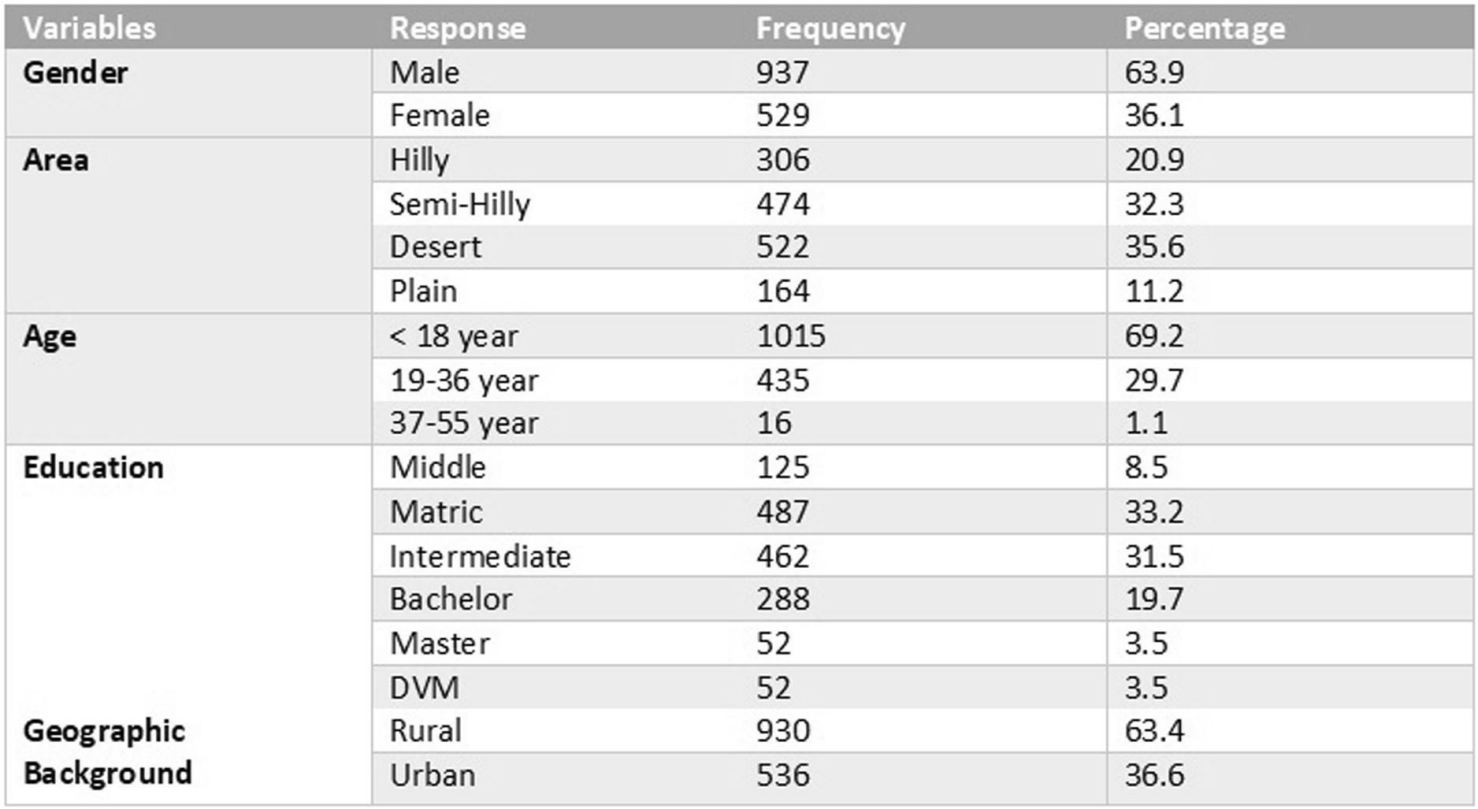
Descriptive Statistics of respondents’ Demographic characteristics

Demographic characteristic of respondents in percentages

### Ethical consideration

This study was approved by the Office of Research, Innovation & Commercialization (ORIC) Institutional Review Committee for Biomedical Research by approval letter No 018/IRC/BMR.

### Questionnaire survey

A questionnaire covering three different sections and twenty three questions was prepared and used for data collection. Section 1 included six questions regarding the participants’ demographic information, Section 2 nine contained questions related to respondents’ knowledge, and Section 3 contained eight questions related with attitude and practices regarding rabies [13].

The structured questionnaire was based on previously conducted KAP surveys on rabies in the world. We sought to include both rural and urban areas representing diverse geographic backgrounds. Section 1 of this study collected demographic details of those different geographic regions that were used as to differences in level of education, age, and gender. In Sections 2 and 3, we included questions designed to access each respondent’s knowledge and awareness regarding rabies disease, mode of transmission, clinical signs, and range of animal host species. Awareness of rabies prevention and control were assessed in relation to attitudes and practices of respondents regarding pre-exposure and post-exposure prophylaxis. Additionally, each respondent’s attitude towards self-vaccination and pet-vaccination were also assessed as an indicator of knowledge towards the prevention and control of rabies. The knowledge and ability to recognize rabid animals were also included in questionnaire in relation to rabies control and prevention strategies.

This survey also included questions of respondent’s knowledge of the conditions of facilities capable of preventative treatment of rabies as a measure to understand and access treatment facilities availability, if someone unfortunately fallen victim to this disease [15]. We also asked respondents about their knowledge and awareness of vaccination and rabies awareness campaigns in their area. Each individual’s attitude regarding pre- and post-exposure prophylaxis (PEP) rabies vaccination was assessed by their willingness to pay for vaccination in order to understand community commitment to eliminate rabies.

### Data analysis

Data was collected by trained survey enumerators to reduce the likelihood of missing critical data points. The participant’s knowledge, attitude, and practices regarding rabies disease were assessed via the structured questionnaire. SPSS 20.0 software was used for initial descriptive analysis and univariate analysis in order to estimate respondent’s knowledge, attitude, and practices related with rabies.

We divided the respondents on the basis of their education level or grade into Middle, Matric, Intermediate level and Bachelor to assess and compare each respondent’s baseline knowledge and awareness of rabies disease.

With our sample size of 1466 individuals, and confidence level of 95%, we were 2.5% within the margin of error of the expected frequency of acceptable knowledge and practices. The associations between rabies patients in near or extended family (outcome/response variable) with all other factors (categorical explanatory variables) were considered significant at *p* < 0.05.

Each respondent’s level of education and their awareness was displayed using frequency table in absolute numbers and percentages. Results from the final models were expressed in terms of odd ratios with associated 95% confidence intervals. There were no known missing values observed within the data collected. Cross tabulation and chi-square analysis with Mantel-Haenszel statistics against study area (Regions) as a layer variable to assess the association between outcome variable and test variables (Table 4). All test variables with a *p*-value of ≤0.20 were included in multivariate analysis to get a clear- idea of an association with a rabies patient in a near or extended-family situation (Table 6). The resulting associations were presented through Venn diagram. We used Microsoft Office 2013 ((Microsoft, Seattle, WA, USA) and Microsoft Excel 2016 (Microsoft, Seattle, WA, USA) in making tables and Quantum GIS Software version 3.6 for making maps.

Chi-square analysis was done to analyze the association of test variables with outcome variable at *p*-value of 0.05. Odds Ratio was also calculated by Mantel-Haenszel Statistics. All factors whose *P*-value was ≤0.20 were included in the multivariate analysis to confirm their true association in the absence of any confounding factor. Model fitting was checked at various levels of significance (Table 6).

## Results

### Demographic characteristics of respondents

A total of 1466 questionnaires were completed from 4 different geographic regions in Punjab and KPK province (hilly region 306 (20.9%), semi-hilly region 474 (32.3%), desert region 522 (35.6%), and plain region 164 (11.2%). More than half of the respondents were from rural regions 930 (63.4%) compared to the respondents from urban areas 536 (36.6%). Rabies related deaths were found to be higher in rural areas 316 (34%) as compared to urban areas (Fig. 3).

The majority of the respondents were males 937 (63.9%) compared to females 529 (36.1%). In our study we intentionally focused on a juvenile age group (< 18 years of age) as this age group is the most frequent victim of a dog bite. A total of 1015 (69.2%) of the data was collected from this juvenile population while 435 (29.6%) and 16(1.09%) of our data was collected from an adult population of over the age of (19–36 years) and (37 to 55 years) respectively (Table 1).

### Respondent’s knowledge about rabies disease

The majority of our respondents 1062 (72.4%) were aware that rabies is a fatal disease. 1152 (78.6%) reported that they knew that an infected dog bite can cause rabies, and 1147 (78.2%) had knowledge about the role of dogs in the spread of rabies. 1024 (69.8%) population were aware that rabies is a vaccine preventable disease. When asked about the best timing of vaccination, 680 (46.4%) responded that rabies vaccine works best before bitten by a dog and 912 (62.2%) responded that a vaccine was more effective if given after a dog bite. This study found 641 (43.7%) of respondents were familiar with the clinical signs of rabies. 431 (29.4%) had rabies victims in their near or extended family. Only 371 (25.3%) had knowledge of any rabies awareness or vaccination campaigns held in their area (Table 2).

**Table 2:**
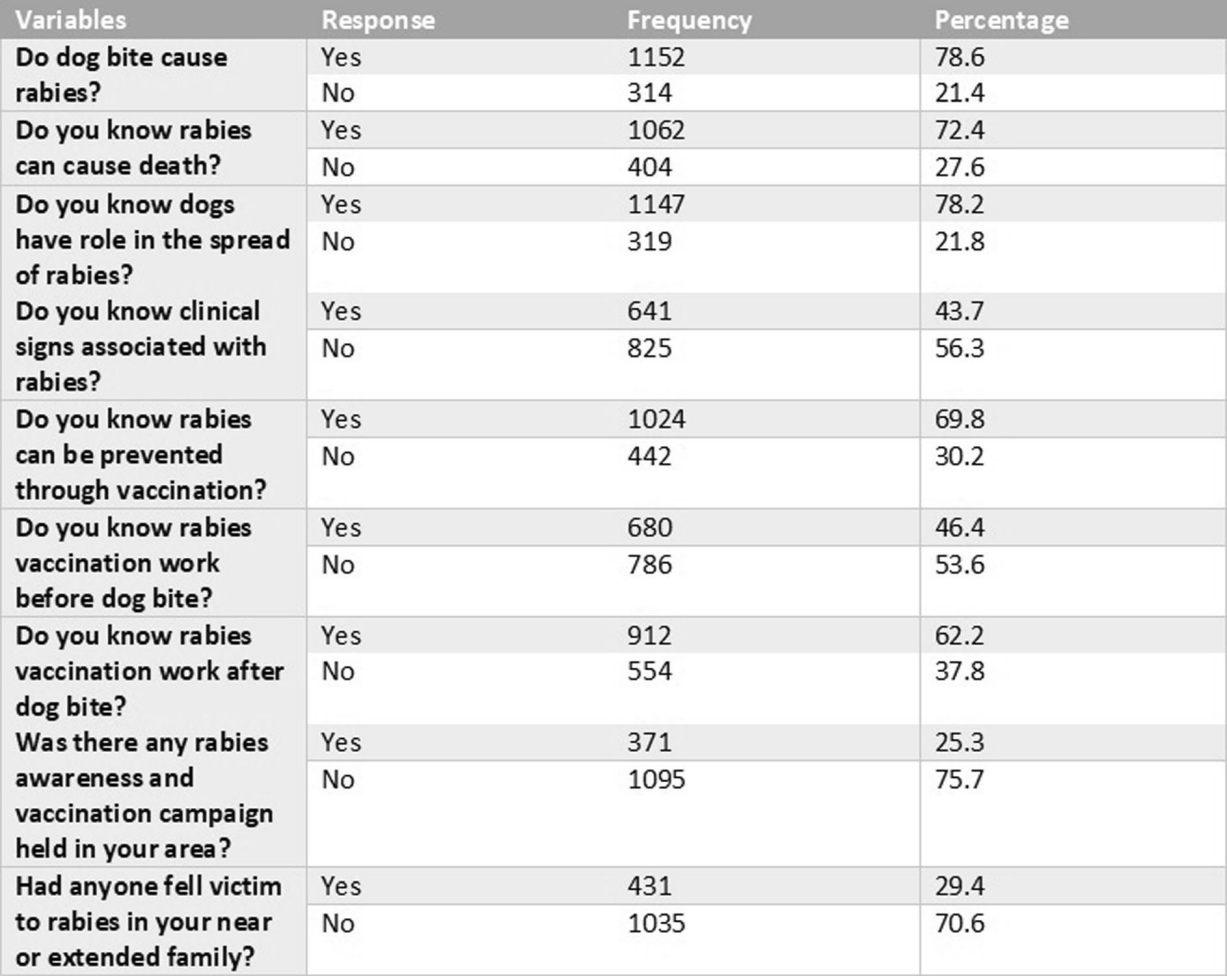
Descriptive Statistics of Respondents’ Knowledge Associated with Rabies Disease

Basic knowledge associated with rabies in percentages

**Fig. 2:**
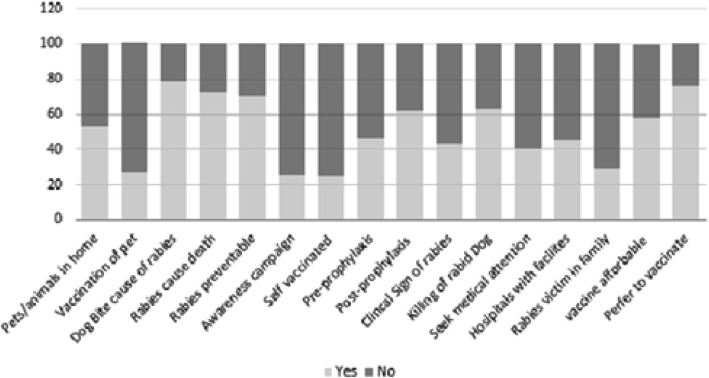
Response Summary of Respondents. Respondents summary of responses of their knowledge, attitude and practices related with rabies disease in percentage. Participents responded in “YES” and “NO” regarding their knowledge, attitude, and practices against rabies diesease

Among all respondents, 1026 (70%) were aware that rabies is a vaccine preventable disease. 407 (77%) of the female respondents were aware that rabies is vaccine preventable disease as compared to 618 (66%) of the male respondents (Table 3). Many 609 (60%) of the juvenile respondents were not aware of the clinical signs associated with rabies, 1056 (72%) of the respondents were aware that rabies was routinely fatal (Fig. 2). More rural respondents 699 (75%) were aware of rabies deadly nature as compared to 363 (67%) of urban respondents.

**Table 3:**
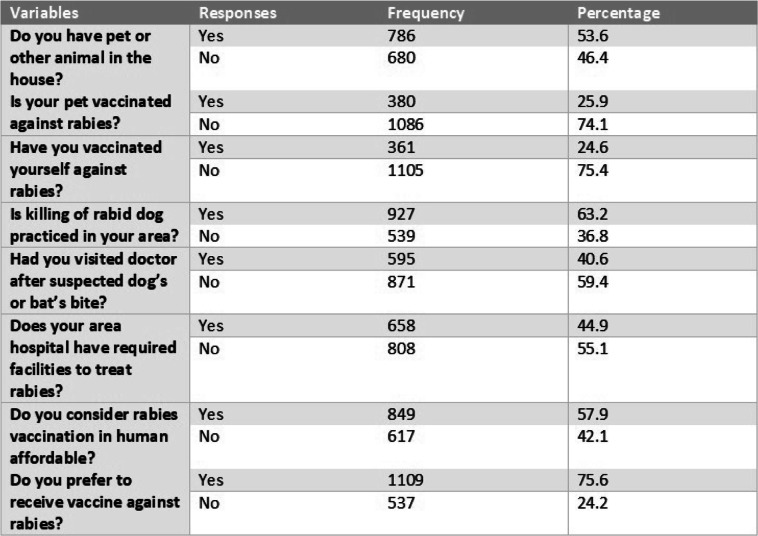
Descriptive Statistics of Respondents’ Attitude and Practices of Respondents regarding Rabies Disease

Attitude and Practices of Respondents regarding rabies in percentages

We assessed education level and its relationship to awareness of rabies, its clinical signs, and practice of seeking medical attention following a suspected dog bite. Our study found that there was a positive relationship between rabies awareness and education level. However, a negative relationship was found in term of level of education and seeking medical attention in case of suspected bite (Table 5).

### Respondent’s attitude and practices about rabies

In this study, 786 (53.6%) of the respondents reported they had animals or pets in their home 380 (25.9%) had vaccinated their animals against rabies (Table 3). The respondents from urban areas 198 (37%) were found to have fewer pets or domestic animals in their homes compared to 595 (64%) of the respondents from rural areas. Although, a large proportion of respondents 1109 (75.6%) reported that they prefer rabies vaccination but only 361 (24.6%) of the respondents actually vaccinated themselves against rabies. It was found that geographic background, pets in the household and pet vaccination rates were significantly associated with each outcome variable (i.e. victim of rabies in near or extended family) (Table 2, Table 4).

**Table 4:**
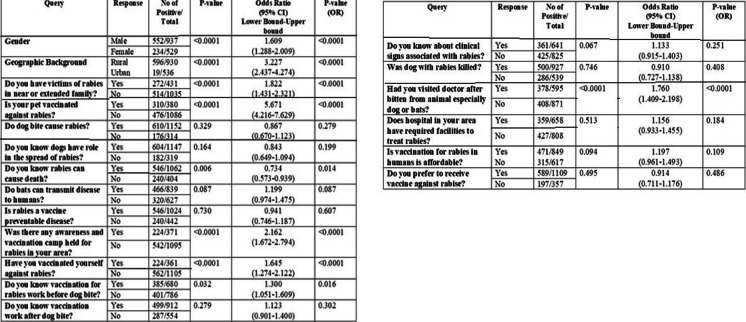
Detailed analysis of the respondent responses regarding Knowledge, attitude and practices of Rabies

No of positive responses, OR ratio and level of significance of the respondent responses regarding knowledge, attitude, and practices related with rabies at 95% confidence interval

When asked about the practice of visiting a doctor after an animal bite (especially dog or bat), only 595 (40.6%) answered they would seek advice from a doctor. 658 (44.9%) of the respondents were aware that their local hospital had the capability to assess an animal bite for rabies. 849 (57.9%) of the respondents considered vaccination against rabies is not affordable (Table 3). 927 (63.2%) of respondent reported that killing of rabid dog is actually practiced in the area (Table 1).

**Fig. 3:**
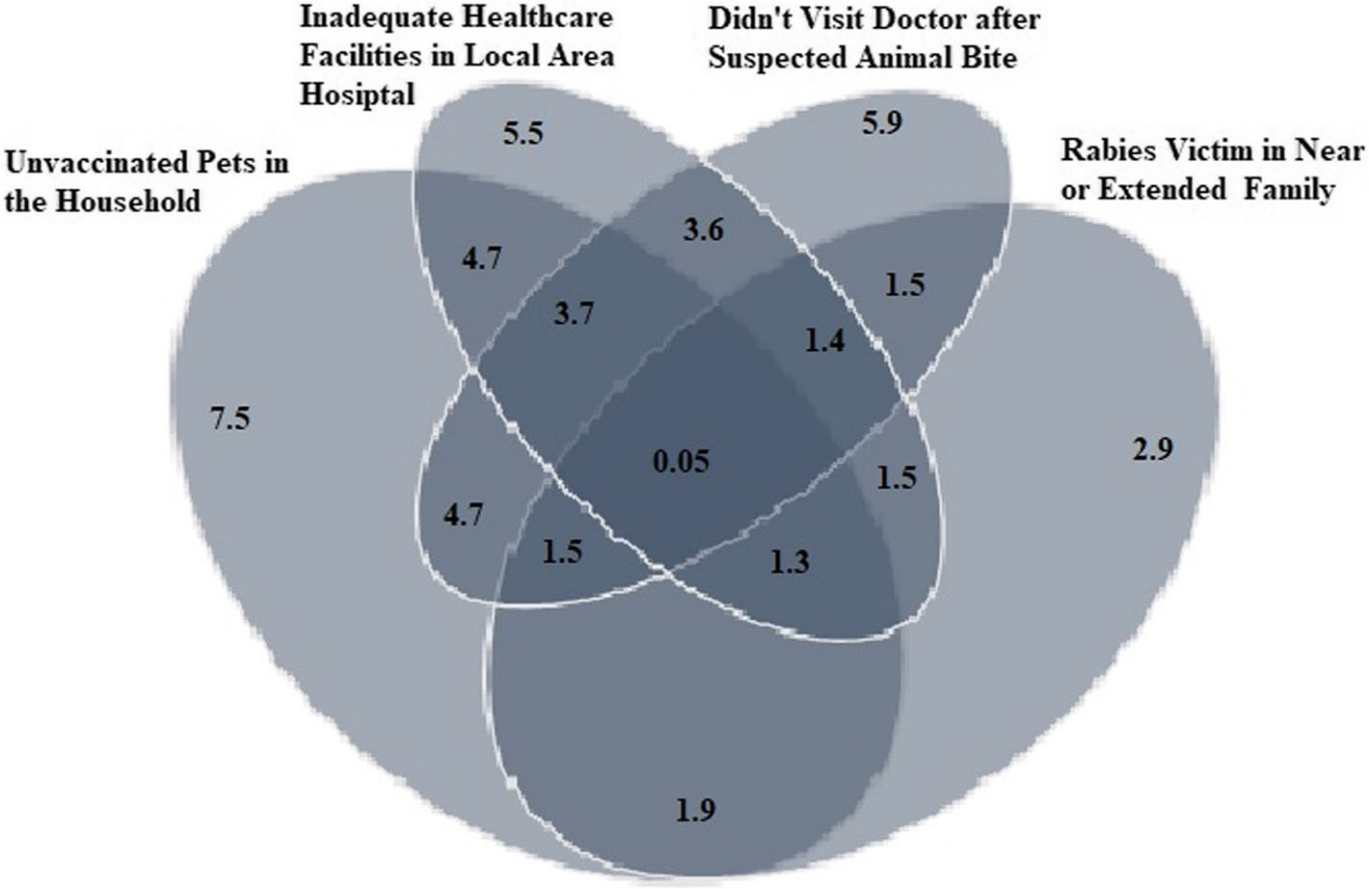
Venn diagram Showing Association of Pets in the Household with Careless Attitudes and Practices of Respondents in Contracting Rabies Disease. Venn Diagram showing association of unvaccinated pets in the household with careless attitude of respondents in not visiting doctor after suspected dog bite, inadequate healthcare facilities in local hospital to manage Rabies patients and resulting deaths in near or extended family of pet’s owner, in proportion

A significant number of the respondents 880 (60%) did not visit a health care professional following a suspected animal bite (Fig. 3). This attitude was found higher amongst 370 (69%) of urban respondents as compared to 502 (54%) of the rural respondents.

### Relative risk exposure of respondents having pets in the household

**Fig. 4:**
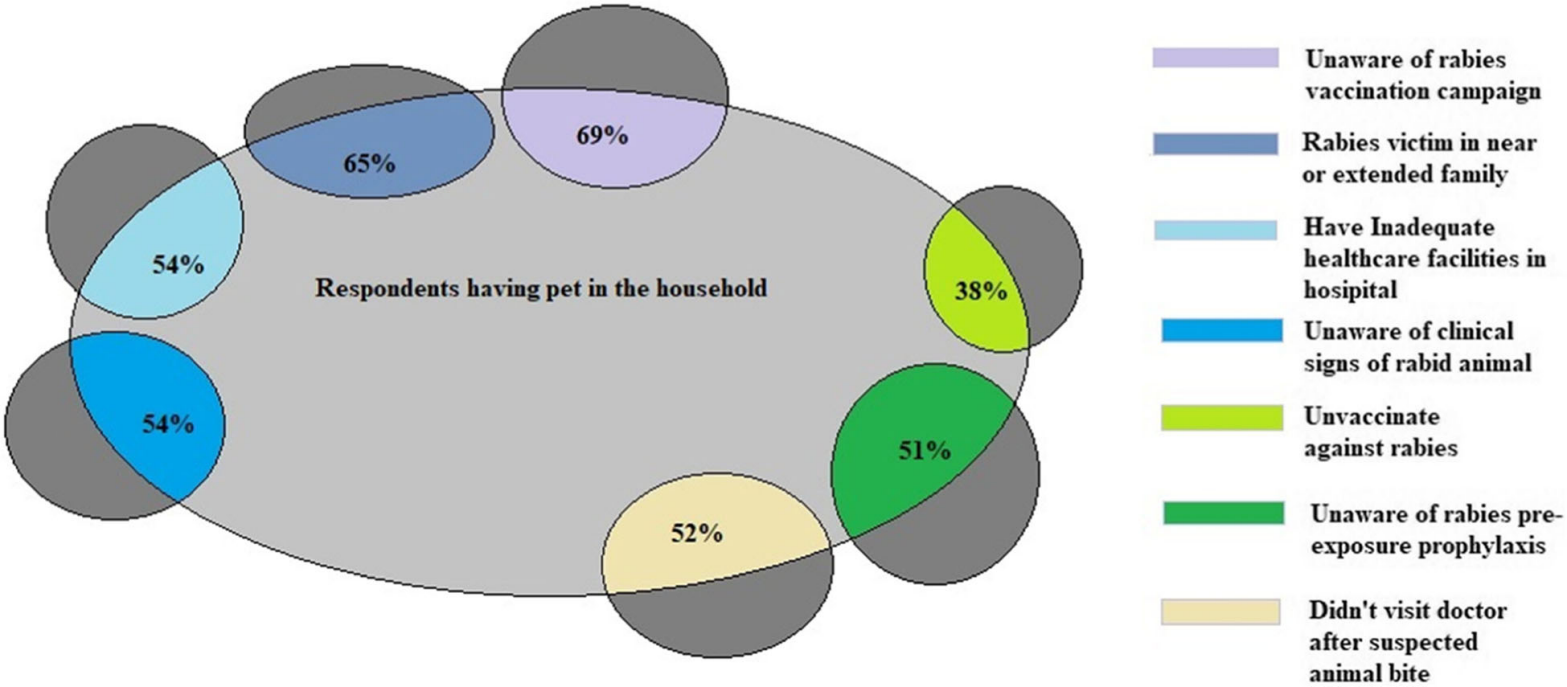
Relative Risk Exposure for Respondents having Pets. Relative Risk Exposure for respondents having pets in comparison with having no pet’s in the household in percentages

Pet owner respondents were unaware of rabies awareness and vaccination campaign in their area [69% (*P* < 0.05; OR 1.96)], haven’t vaccinated [38% (*P <* 0.05; OR 1.58)] themselves against rabies due to less awareness of pre-exposure prophylaxis [51%; (*P <* 0.05; OR 1.25)] had not sought medical advice after a suspected animal bite [52% (*P <* 0.05; OR 1.97)] or had resulted in more deaths of someone in their near or extended family due to rabies [65%; (*P <* 0.05; OR 1.73)]. Similarly pet owners were comparatively at greater risk for contracting rabies due to their lack of knowledge and awareness of the various clinical signs of rabies in animals [54%, (*P <* 0.05; OR 1.20)] to those having no pets in the household (Tables 2, 3, 4, 5 and 6, Fig. 4).

**Table 5:**
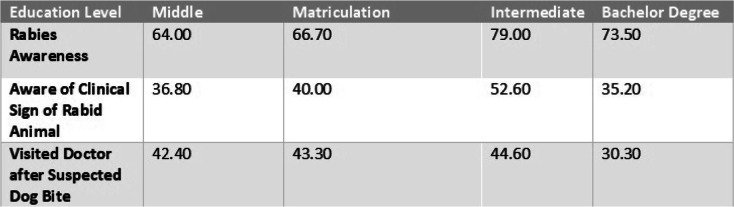
Cross tabulation of respondents’ Education Level with Rabies Awareness and Clinical Sign of Rabid Animal and Practice of Seeking Doctor after Suspected Animal bite

Relationships of education level with rabies awareness and clinical sign of rabid animal and practice of seeking doctor after suspected animal bite in percentages

**Table 6:**
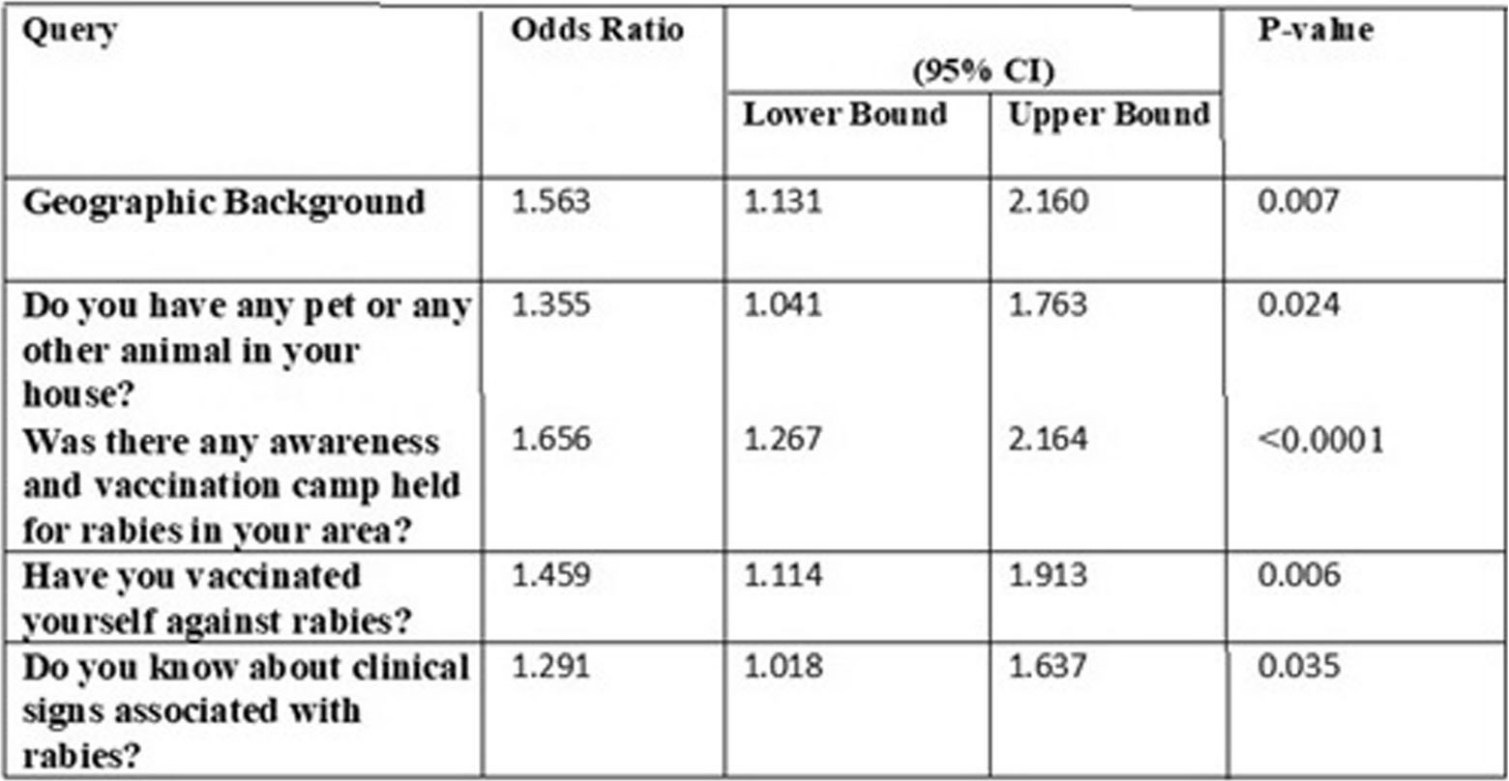
Multivariate Analysis for those variable having association with Rabies patient

All those variables with a *P*-value of ≤0.20 were included in multivariate analysis to get a clear-cut idea of association with rabies patient in near or extended family

## Discussion

Rabies remains an important global public health problem particularly in developing countries such as Pakistan. Since rabies is regarded as a neglected tropical disease, limited general public knowledge and awareness campaigns are conducted across the globe. Recent work from WHO under the umbrella of “Zero Rabies by 2030” have resulted in many countries have started efforts to minimize the risk of rabies due to dog bite [14, 16]. Keeping in view the limited knowledge of the local community, we initially assumed that approximately 50% study population would have the basic knowledge and required awareness regarding clinical signs, transmission and preventive measures to control dog bite associated rabies. We also initially assumed that they can adopt suitable practices in their life to avoid any contact with rabid animal or rabies based upon that basic knowledge of rabies. In this KAP study of 1466 subjects, our findings are within 2.5% margin of error of the expected frequencies of acceptable knowledge and practices. Our results highlight key findings regarding the level of awareness in people considered at high risk for contracting rabies.

Gender bias was not found to be a potential risk factor for rabies victims in near or extended family members. We found that the geographic region is a strong risk factor for rabies deaths. This might be due to the fact that in rural areas, no vaccination or awareness campaigns exist and dogs are frequently found to be free roaming in streets [17]. Dogs are not routinely vaccinated in these areas, increasing the risk of transmission of rabies from animals to humans. Previous studies suggested that children less than 18 year of age are more prone to rabies and dog bite compared to adults [14],

The majority of respondents of this survey had pets or domestic animal in their household similar to other parts of the world [11]. We found that most of those respondents did not vaccinate their pets against rabies. This finding was consistent with findings of prior KAP surveys in India, Ethiopia, and Grenada [15, 17, 18]. This is a very alarming situation as animals are the main source of disease transmission to humans [15, 17]. We also observed that many of our respondents were not aware of rabies disease and its deadly nature despite many of them being aware of the clinical signs associated with rabies is a finding similar to previous studies in the Philippines, Bangladesh, and Tanzania [19–21].

One of the critical findings of this survey is that the majority of the respondents revealed that they did not seek urgent medical care following a dog bite, consistent with similar studies on rabies in Pakistan [22]. However, this is in contrast to previously published studies on rabies in developed nations across the globe [11, 13, 18, 23]. All these factors indicate that population in the study area are at a constant risk of disease because the participants lack sufficient knowledge regarding potential source, prevention, and control of rabies.

Two-thirds of our respondents had little knowledge regarding awareness of vaccination activity and campaigns against rabies in their region. This finding is significant in rabies control and elimination efforts. Awareness of rabies, in term of its etiology, route of transmission, major hosts, and reservoir is expected to result in reduced number of rabies cases in Pakistan [24]. This awareness can result in actionable measures such as change in attitude of the people who have close contact with their unvaccinated animals, particularly dogs. Their attitude toward dog bite and subsequent wound management can significantly improve if they are aware of the risks associated with it. Wound management after a dog bite is a significant step to prevent rabies disease. Unfortunately, many of the respondents in our survey lacked sufficient knowledge about the importance of this practice. Improper wound management instantly after a dog bite and seeking no medical attention inevitably results in death if the animal is rabid, which could be prevented through this essential step in Pakistan.

Increasing knowledge and awareness of people particularly of rural background is vital for efforts to control and eliminate rabies in developing countries including Pakistan. The common source of information about rabies is disseminated through personal contact, media (television, radio and newspapers) and from professionals such as health workers, researchers (during their research activities), or teachers at school [19]. Pakistan rabies awareness program is still far behind and it requires utilization of all of these channels rather than disseminating rabies awareness through illustration charts and posters in state hospitals.

The majority of respondents of this survey had heard about rabies as a vaccine preventable disease and most were aware of its transmission through dog bites. These result were comparable to the KAP surveys conducted in regional countries [11, 25] but respondents in this survey had less knowledge regarding post-exposure prophylaxis and the majority were uncertain regarding any rabies awareness or rabies vaccination campaigns in their area.

The majority of our respondents did not seek hospital care after a dog bite compared to 90% of people in Bhutan, Tanzania, Sri Lanka, and Ethiopia [11, 18, 19, 25]. This attitude and practice could be seen as significant factor in the number of deaths associated with rabies in Pakistan.

It is also observed some people seek traditional remedies and spiritual healers to cure rabies instead of visiting hospitals. This practice of seeking a spiritual healer for possible rabies patient is also reported in Africa and India [26, 27]. Although we did not specifically look into this issue in the survey, this might also explain why our study demonstrated 60% of the respondents did not seek traditional medical attention. They might have sought ought spiritual healers, many of which offer treatment free of cost. It is for this reason that we should ultimately consider it prudent and necessary to include spiritual healers in our efforts to eliminate rabies in Pakistan. These spiritual healers and leaders may very well serve as an effective resource individual and community leader if we provide incentives to them and can also help to assess the true burden of rabies in Pakistan [20].

Another explanation for not seeking doctor among bite victims is that post-exposure prophylaxis (PEP) costs from 1600 to 2400 (9.55 to 14.32 USD) Pakistani Rupee per vial, the equivalent of 2 day salary for an average resident. That cost is mostly paid by the patient due to the general lack of availability of the rabies vaccine in government hospitals, which results in lack of adherence to medical recommendations. Future studies should further explore the reasons for lack of PEP adherence, with attention to issues related to insurance coverage, costs to the patient, and perceived risk. WHO guidance recommends monoclonal antibody cocktails to fill critical gaps in PEP availability in countries in such condition. Monoclonal antibodies can be evaluated as a potential solution for this apparent unavailability of PEP [14, 28].

One of our major study limitations is the sampling method and study area covered because these results cannot be extrapolated to all of Pakistan. Another caveat is absence of a scoring system, which makes it difficult to fully assess overall picture. Our study did not have sufficient financial resources to cover Sindh and Baluchistan provinces as well as other federally administered territories. Some of these areas especially in Sindh Province were increasingly reporting rabies related human mortalities due to lack of vaccine and inadequate health care facilities in local hospitals. Many other regions in Pakistan are data deficient in term of rabies related mortalities. Therefore, this study recommends covering all areas and regions for additional specific data of rabies disease in all areas of Pakistan.

The main strength of this study is its timeliness. This study is the first study that has covered a wide geographic area with a large sample size in Pakistan. Hopefully, this study will lead to future studies designed to reduce the number of rabies related mortalities in Pakistan. Rabies is increasingly claiming deaths which generate public outcry, mostly due to painful death but also due to unavailability of rabies vaccine. Pakistan has poor health care infrastructure, as it is seen that there are only two rabies management centers in metropolitan city of Karachi in Sindh Province, moreover, these centers also serve people from the interior of Sindh and Baluchistan province, a vast area of southern Pakistan. These centers record over 20,000 dog bites cases annually [29].

This KAP Survey also helps to target our efforts to improve health-seeking behavior like post exposure prophylaxis and seeking medical attention after dog bite. The finding of this survey will hopefully improve local rabies knowledge, attitudes, and practices across the study region and ultimately to the entire population of Pakistan. There is a dire need to invest in upgrading health care infrastructure and rabies disease surveillance for effective control and prevention of rabies in Pakistan.

## Conclusions

We identified a significant need to increase awareness of rabies through vaccination campaigns, community meetings, and information disseminated through media. These measures can be effective to improve human attitudes towards proper wound management and seeking medical attention after a dog bite. Mass dog vaccination is the best way to control rabies and to prevent human deaths. However, this would be challenging, in terms of cost and technical expertise [30] in Pakistan. Implementation of rabies legislation in Pakistan is likely to be effective only if it is implemented hand-in-hand with increased accessibility of affordable interventions, such as primary health care and vaccination. An experience learnt from other infectious diseases show that these policies of subsidizing these interventions bring positive results in the control and prevention of infectious disease like rabies. These finding of study could help in devising rabies disease policy and targeted management strategies to prevent rabies related deaths in Pakistan.

## Supplementary information

**Additional file 1.** Questionnaire. The questionnaire used in this study.

**Additional file 2.** Datasheet. The excel datasheet used in this study.

## Data Availability

The datasets generated during and analyzed during the current study are available in the Dryad repository, https://datadryad.org/stash/share/1X3c55Dy19WAebAxmWZqxBKUpNpcCX_cvltJ_4BI_DQ

## Abbreviations

KAP: Knowledge, Attitude and Practice
P: Probability
OR: Odd Ratio
KPK: Khyber Pakhtunkhwa
