# Supplementary material for: Knowledge, attitude and practice (KAP) survey of canine rabies in Khyber Pakhtunkhwa and Punjab Province of Pakistan
Source: BMC Public Health. 2020 Aug 26;20:1293. doi: 10.1186/s12889-020-09388-9 (PMC7448460; doi:10.1186/s12889-020-09388-9)
Supplement: Supplementary file 1 — Additional file 1. Questionnaire. The questionnaire used in this study. [file 12889_2020_9388_MOESM1_ESM.pdf]

Dr. Touseef Ahmed (DVM. R.V.M.P, M.V.P.H Scholar). Department of Epidemiology and Public Health

Knowledge, Attitudes and Practices (KAP) Survey of Rabies Disease, Pakistan

Name____

Area (district)___________________________Education_________________________________

Age (years) A. Less than 18 B. 19---36 C. 37------55 D. 55---above

Gender	Male	Female
Geographical Background	Rural	Urban
Do you have any pet or any other animal in your house	Yes	No
Is your Pet vaccinated against rabies	Yes	No
Do Dog bite causes rabies	Yes	No
Do you know rabies can cause death	Yes	No
Do you know dogs have role in spread of rabies	Yes	No
Is rabies vaccine preventable Disease	Yes	No.
Was there any awareness & vaccination camp held for rabies in your area	Yes	No
Have you vaccinated yourself against rabies	Yes	No
Do you know vaccination for rabies work before dog bite	Yes	No
Do you know vaccination work after dog bite	Yes	No
Do you know about clinical sign associated with rabies	Yes	No
Was dog with rabies killed	Yes	No
Had you visited doctor after bitten from animal especially dogs or bats	Yes	No
Does Hospital in your area have required facilities to treat rabies	Yes	No
Do you have any one suffer from rabies in your family	Yes	No
Is vaccination for rabies in human is affordable	Yes	No.
Do you prefer to receive vaccine against rabies	Yes	No.
